# Excision of the Lateral Half of the Tongue

**Published:** 1871-10

**Authors:** 


Excision of the Lateral Half of the Tongue.—Dr. George Buchanan reports three cases operated upon by him in the Glasgow Infirmary for chronic disease of the tongue simulating cancer. An incision in all three cases was made through the center of the lower lip down to the hyoid bone, then the lower jaw was sawn through; a piece of string was next passed around the divided sides to hold the jaw apart. The muscular attachments of the tongue were severed and the organ was drawn well forward. An incision was then made down the center of the tongue to the base, and then by a slight curve of the knife, the remaining attachments of the affected side were severed and it was removed. Hemorrhage was promptly checked by tying the lingual artery. The wound was sponged with a solution of chloride of zinc, and the jaw was held in place by boring a hole through each side and passing through a silver wire and twisting it off. In all of the cases the speech was quite intelligible after recovery.—*Ed. Medical Journal.*

